# In Situ Agarose Microfabrication Technology Using Joule Heating of Micro Ionic Current for On-Chip Cell Network Analysis

**DOI:** 10.3390/mi13020174

**Published:** 2022-01-25

**Authors:** Kenji Shimoda, Haruki Watanabe, Yoshitsune Hondo, Mitsuru Sentoku, Kazufumi Sakamoto, Kenji Yasuda

**Affiliations:** 1 Department of Pure and Applied Physics, Graduate School of Advanced Science and Engineering, Waseda University, Tokyo 169-8555, Japan; kenji.s@toki.waseda.jp (K.S.); watanabeharuki@akane.waseda.jp (H.W.); hondoh@asagi.waseda.jp (Y.H.); mitsen1019@fuji.waseda.jp (M.S.); sk.060603@akane.waseda.jp (K.S.); 2 Department of Physics, School of Advanced Science and Engineering, Waseda University, 3-4-1 Okubo, Shinjuku, Tokyo 169-8555, Japan

**Keywords:** agarose microfabrication, Joule heating, micro ionic flow, tapered microcapillary tube, constructive cell network analysis

## Abstract

Agarose microfabrication technology is one of the micropatterning techniques of cells having advantages of simple and flexible real-time fabrication of three-dimensional confinement microstructures even during cell cultivation. However, the conventional photothermal etching procedure of focused infrared laser on thin agarose layer has several limitations, such as the undesired sudden change of etched width caused by the local change of absorbance of the bottom surface of cultivation plate, especially on the indium-tin-oxide (ITO) wiring on the multi-electrode array (MEA) cultivation chip. To overcome these limitations, we have developed a new agarose etching method exploiting the Joule heating of focused micro ionic current at the tip of the micrometer-sized capillary tube. When 75 V, 1 kHz AC voltage was applied to the tapered microcapillary tube, in which 1 M sodium ion buffer was filled, the formed micro ionic current at the open end of the microcapillary tube melted the thin agarose layer and formed stable 5 μm width microstructures regardless the ITO wiring, and the width was controlled by the change of applied voltage squared. We also found the importance of the higher frequency of applied AC voltage to form the stable microstructures and also minimize the fluctuation of melted width. The results indicate that the focused micro ionic current can create stable local spot heating in the medium buffer as the Joule heating of local ionic current and can perform the same quality of microfabrication as the focused infrared laser absorption procedure with a simple set-up of the system and several advantages.

## 1. Introduction

The importance of geometric arrangements of cellular networks has been unveiled with advancements of microfabrication technologies [1,2,3]. One of the two major approaches to form the desired confined spatial arrangement of cells is micro etching technology. It is the etched microstructures based on the semiconductor etching with photolithography or the three-dimensional polymer structures formed with cast-mold method [4,5]. The advantage of this micro etching technology is strict and precise physical control of their microstructures to arrange cells’ positions and connections in these spaces of three-dimensional microstructures for assessing cell behaviors. However, the pre-designing of photomasks and complicated exposure-development procedures are required for this approach. Especially, the process of alignment adjustment of photomasks requires experienced techniques and expensive machines. Moreover, the designed structures were fixed before cell cultivation started, and no changes were allowed. To overcome these limitations, recently, several methods for maskless fabrication, such as the direct, focused two-photon excitation or exploiting the microfluidic flows, have been proposed [6].

In contrast, the other microprinting technology is simple stamping of adhesion factors on the flat substrate to control the spatial distribution of cells [7,8]. Adding to the simplicity, stepwise activation of stamped thermoresponsive molecules for direct neurite elongation in pre-designed patterns during cultivation was also recently reported [9].

However, in both technologies, the designs are pre-determined by the photomasks or stamps and thus render difficulty preparing changes in complex structures and additional micropatterning after the cells are seeded onto the micro-engineered environment.

To overcome the above limitations of two conventional major microfabrication technologies, we have developed another microfabrication technology based on agarose photothermal etching. This technique can create micrometer-sized engineered structures by melting a portion of the thin agarose layer placed on the cultivation dish with spot heating of focused infrared laser, regardless of whether the cells are present or not, without any photomasks nor complicated development-exposure procedures [10,11,12,13,14]. Moreover, improving the advantages of this flexible agarose microfabrication method, we have accomplished the spatial resolution of agarose etching beyond the diffraction index limit of the focused infrared laser wavelength, and also indicated this technology can perform additional microfabrication processing during neuronal cell cultivation to guide each neurite to the desired direction, which was difficult using existing methods [15]. Although the continuous improvements have brushed up the unique advantage of agarose microfabrication technology against the conventional microfabrication technologies, it remained the difficulties for etching agarose layer on the multi-electrode array (MEA) chip because of the difference of absorbance of indium-tin-oxide (ITO) wires on the substrate.

Here, we report a new approach of agarose microfabrication technology using Joule heating of micro ionic current instead of the conventional direct spot heating of focused infrared laser. As this new approach is independent of the absorbance of medium buffer and the material of the cultivation dish, it has the potential to overcome the limitations and complexity of the focused laser-based agarose photothermal microfabrication technology. The ability and performance of this new technology have been evaluated and found that the resolution and stability of this new approach are equal or better than the photothermal method only with the simple set-up of AC power supply and glass microcapillary tube.

## 2. Materials and Methods

### 2.1. Agarose Microfabrication System by Using Joule Heating of Micro Ion Current

The system for agarose microfabrication using Joule heating of micro ionic current consists of four components; a platinum-electrode-inserted tapered glass microcapillary tube (King Precision Glass Inc., Claremont, CA, USA) attached to a three-dimensional micromanipulator (MHW-3, NARISHIGE, Tokyo, Japan), a motorized x-y stage (BIOS-206T, SIGMA KOKI, Tokyo, Japan) for placing and moving the cultivation dish with 0.2 μm resolution, a function generator (33120A, Hewlett-Packard Co., Palo Alto, CA, USA) connected to a highspeed bipolar amplifier (BA4825, NF Co., Kanagawa, Japan) for applying AC voltage, and a phase-contrast microscope (IX-71 with ×20 phase-contrast objective lens, LCUPLFLN, OLYMPUS, Tokyo, Japan) equipped with a CMOS camera (DCC 3260C, Thorlabs Inc., Newton, NJ, USA) for observing the processing.

The tip of the tapered glass microcapillary tube was fabricated by a micropipette puller (P-97, Sutter Instrument Co., Novato, CA, USA) to an outer diameter of 3 μm. The glass microcapillary tube was filled with 1 M NaCl solution as the electrolyte solution. Platinum wires (PT-B, NARISHIGE, Tokyo, Japan) were used for electrodes inserted in the glass microcapillary tube and for the reference electrode.

A 35 mm tissue culture dish (3000-035, AGC TECHNO GLASS Co., Ltd., Shizuoka, Japan) was hydrophilized by plasma ion bombarder (PIB-20, Vacuum Device, Ibaraki, Japan) and coated with 2.5% agarose (agarose low melting point analytical grade, v2111, Promega, Madison, WI, USA) by a spin coater (1H-D7, MIKASA, Tokyo, Japan). Finally, cultivation medium was added to the agarose-coated cultivation dish for agarose microfabrication.

A multi-electrode array (MEA) chip (MED-P5155, AlphaMED scientific Co., Ltd., Osaka, Japan) was prepared same procedure as the 35 mm tissue culture dish.

### 2.2. Resistance Measurement of Glass Capillary Tube

Resistance of microcapillary tube was measured by a patch-clamp microelectrode amplifier (MultiClamp 700B, Molecular Devices LLC, San Jose, CA, USA) and a digitizer (Digidata 1440A, Molecular Devices LLC, San Jose, CA, USA). For the measurement, 1 M NaCl was chosen as bath solution in a cultivation dish and was also filled into the glass microcapillary tube. Then, a pulsed voltage was applied to bath solution, and the resistance was estimated using the analysis software, Clampex (Molecular Devices LLC, San Jose, CA, USA). The mean value and standard deviation were acquired from the resistance values of 12 glass microcapillary tubes.

### 2.3. Heat Distribution Measurement

The heat distribution in the glass microcapillary tube was measured by the fluorescence intensity change of rhodamine B (Sigma-Aldrich, St. Louis, MO, USA) solution in the capillary tube. The composition of Rhodamine B solution is 100 μM Rhodamine B and 1 M NaCl. Fluorescence quenching was observed with a cooled charge-coupled-device camera imaging system (ORCA-ER, Hamamatsu Photonics, Shizuoka, Japan). Fluorescence intensity was measured by ImageJ (US National Institutes of Health, Bethesda, MD, USA).

### 2.4. Confocal Cross-Sectional Imaging of Agarose Layer

Cross-sectional images of agarose layer were acquired with confocal microscopy (FLUOVIEW, Olympus, Tokyo, Japan). For the fluorescence imaging, the agarose layer was stained with 0.05 μm fluorescent microspheres (Fluoresbrite YG, Polysciences, Inc., Warrington, PA, USA).

### 2.5. Preparation of Collagen-Coated Cultivation Dish

A collagen-coated 35 mm dish was prepared with Cellmatrix Type 1 -C (KP-4100, Nitta Gelatin Inc., Osaka, Japan) diluted to 10-folds with one mM HCl. 200 μL of the collagen solution was spread on a tissue culture dish evenly and dried up. Then, the dish was coated with 2.5% agarose by a spin coater. The agarose-coated dish was washed three times with ultrapure water, co-washed once with culture medium, and filled with 2 mL of culture medium.

### 2.6. Cell Cultivation

This study was carried out in strict accordance with the Act on Welfare and Management of Animals of the Ministry of the Environment, Japan. All animal experiments and protocols were approved by the Animal Experiment Committee of Waseda University (permission number: 2021-A087) and adhered to guidelines (ARRIVE 2.0) and regulations for animal experimentation.

Embryonic mouse primary cardiomyocytes were isolated and purified from 14-day-old ICR mouse (Tokyo Laboratory Animals Science Co., Ltd., Tokyo, Japan) embryos according to a modified version of the method described in our previous reports [16,17].

Cardiomyocytes were isolated and purified with 0.25% collagenase (Wako Pure Chemical Industries Co., Osaka, Japan) diluted by PBS (Takara Bio Inc., Shiga, Japan) and pre-cultured for two days at 37 °C with 5% CO_2_ in primary cultivation buffer, consisting of Dulbecco’s modified Eagle’s medium (DMEM: Invitrogen, Carlsbad, CA, USA) supplemented with 20% heat-inactivated fetal bovine serum (FBS: Invitrogen, Carlsbad, CA, USA), 100 U/mL penicillin and 100 μg/mL streptomycin (Invitrogen, Carlsbad, CA, USA), on a 35 mm tissue culture dish coated with Cellmatrix Type 1-C (KP-4100, Nitta Gelatin Inc., Osaka, Japan) diluted 10-fold with 1 mM HCl.

Afterward, the cardiomyocytes were re-dispersed by 0.25% trypsin EDTA (25200056, Invitrogen, Carlsbad, CA, USA) and placed one by one into the microchamber by using a heat-polished glass pipette (GD-1, NARISHIGE, Tokyo, Japan).

Rat hippocampal neurons were isolated and purified from 18-day-old Wister rat embryos (Tokyo Laboratory Animals Science, Tokyo, Japan) using Neuron Dissociation Solutions (FUJIFILM Wako Pure Chemical, Osaka, Japan). The hippocampal neurons were cultured within Neuron Culture Medium (FUJIFILM Wako Pure Chemical) in the agarose-patterned-poly-d-lysine (Sigma-Aldrich, St. Louis, MO, USA) coated dish at 37 °C in 5% CO_2_ at saturated humidity. The neurons were placed, one by one, into each round microchamber in the agarose with a fire-polished glass pipette.

Cardiomyocytes and neurons were observed using a phase-contrast microscope equipped with a CMOS camera (DCC3240C, Thorlabs Inc., Newton, NJ, USA).

### 2.7. Statistical Analysis

All statistical values of widths and lengths are presented as mean ± standard deviation (S.D.) of ten points measurements (unless stated otherwise).

## 3. Results and Discussion

### 3.1. Agarose Microfabrication with Joule Heating of Micro Ionic Current

In this study, we have examined the new approach of agarose microfabrication technology utilizing the Joule heating of micro ionic current for spot melting of the portion of agarose layer coated on the bottom surface of cultivation dish. As the Ohm law of electric circuits can be applied to the ionic current in the medium, we used this principle for melting of thin agarose layer.

Joule heating or Ohmic heating is well known as the heating generated by an electrical conductor and is proportional to the product of its resistance and the square of the electric current,

(1) $$P=IV=I^{2}R=\frac{V^{2}}{R},$$

where *P* is the power converted from electrical energy to thermal energy, *I* is the electric current, *V* is the applied voltage drop across the heating element, and *R* is the resistance of the element. In this experiment, we adapted this law to the micro ionic current to generate localized heat around the focused ionic current.

As the micro ionic current is a microfluidic flow, we should consider the behavior of this current satisfying continuity equation $\nabla \;\cdot \;\vec{I}=0$ according to the geometric change of the microfluidic pathway. For example, when we use the tapered microcapillary tube to focus the micro ionic current at the discharge opening (tip of the tapered microcapillary tube), the intensity of ionic current has been increased geometrically,

(2) $$I_{tip}={\left(\frac{D_{0}}{D_{tip}}\right)}^{2}I_{0},$$

where $I_{tip}$, $D_{tip}$ and $I_{0}$, $D_{0}$ are the micro ionic currents and inner diameters of holes at the tip of the tapered microcapillary tube and at the position of the electrode set in the capillary tube, respectively. Hence, the product of heating caused by the micro ionic current in the tapered microcapillary tube $P_{tip}$ is described as

(3) $$P_{tip}=IV=I^{2}R={\left(\frac{D_{0}}{D_{tip}}\right)}^{4}I_{0}^{2}R,$$

This equation indicates that the tapered ionic current with geometric confinement can generate area-specific heating with the fourth power correlation of tapered geometry. Hence we applied a tapered microcapillary tube to form a micrometer-sized spot heating at the tip of the capillary tube for agarose microfabrication.

**Figure 1:**
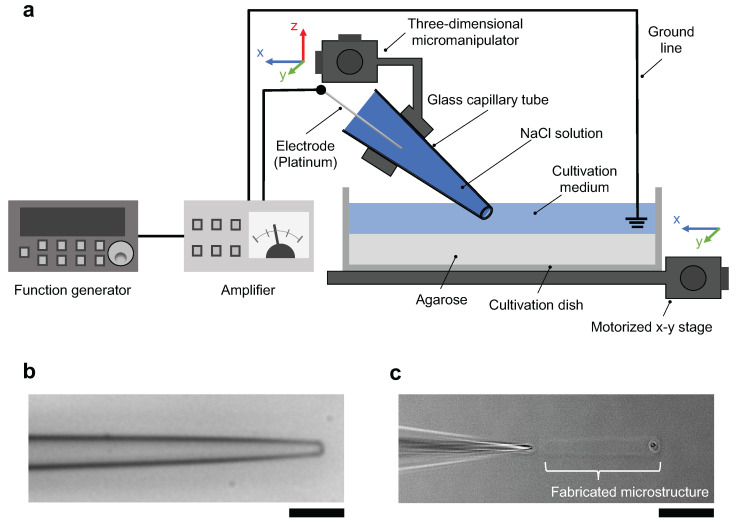
Micro ionic current agarose microfabrication system. (**a**) Schematic drawing of the micro ionic current agarose microfabrication system. The tapered glass microcapillary tube was filled with 1 M NaCl solution. The cultivation dish was filled with cultivation medium. The AC output of the function generator and amplifier was applied to a glass microcapillary tube, and the formed ionic current passed through the 2–3 μm open end of the microcapillary tube. The Joule heating of focused micro ionic current at the tip of the microcapillary tube melted the agarose layer coated on the bottom surface of the cultivation dish. The agarose structures were designed by the movement of 0.2 μm-resolution motorized x-y stage. (**b**) Bright-field image of the tapered glass microcapillary tube. The outer diameter of the tip of the tube was 2.6 μm. Bar, 10 μm. (**c**) Phase-contrast image of agarose microfabrication with Joule heating of micro ionic current. A portion of the agarose layer was melted by Joule heating of focused micro ionic current at the tip of glass microcapillary tube. According to the movement of the x-y stage from left to right, the agarose microchannel was formed in the agarose layer. Bar, 30 μm.

The schematic drawing of the micro ionic current agarose microfabrication system is described in Figure 1a. The system consists of the following four major parts; an agarose-coated cultivation dish filled with cultivation medium set on a motorized x-y stage, a tapered glass microcapillary tube filled with 1 M NaCl solution in which a platinum wire was inserted, a three-dimensional micromanipulator to allow fine adjustment of the position of the glass microcapillary tube tip, and a function generator and an amplifier to apply AC voltage between the microcapillary tube and the platinum wire ground line set in the cultivation dish. When the AC voltage was applied to the microcapillary tube, a focused micro ionic current was generated at the tip of the tapered microcapillary tube. Finally, Joule heating of micro ionic current at the open end of the glass microcapillary tube melted a portion of an agarose layer.

Figure 1b shows a bright-field image of the tip of the glass microcapillary tube. The outer diameter of the open end is 2.6 μm, which is comparable to that used in the whole-cell patch-clamp method. The resistance was measured with 12 tubes using 1 M NaCl solution, and the mean ± SD of the resistance was 2.5 ± 0.97 MΩ.

Figure 1c shows a phase-contrast image of a linear structure (microchannel) being fabricated. Fabrication was proceeded from the right side to the left side of the image by moving the motorized x-y stage with the glass microcapillary tube fixed on top of the agarose layer.

### 3.2. Fabrication Direction Dependence of Micro Ionic Current Method

In the micro ionic current method, Joule heating was formed by the directed flow of ionic current from the open end tip of the tapered microcapillary tube. Hence the etched agarose shape might depend on fabrication direction and microcapillary tube direction, parallel to the ionic current direction. If so, we need to consider the microcapillary tube direction for precise etching of the agarose layer. Therefore, we examined the fabrication direction dependence, horizontal and vertical, to the micro ionic current.

**Figure 2:**
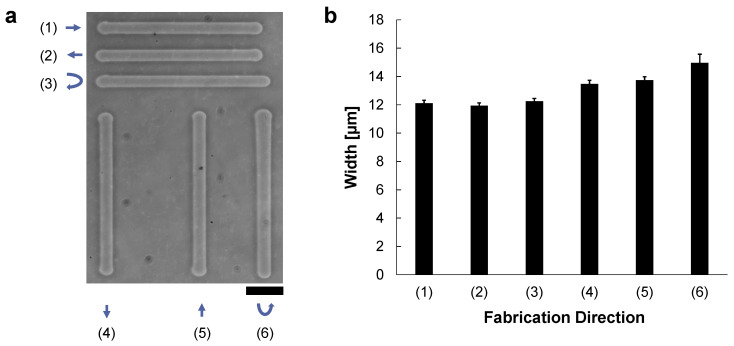
Direction dependence of the micro ionic current agarose microfabrication. These agarose microchannels were fabricated at the 90 V, 1 kHz AC condition. The microcapillary tube was set horizontally from left, and micro ionic flow was from left to right direction. The movement speed of the motorized stage for etching lines was 10 μm/s. (**a**) A phase-contrast image of formed agarose microchannels in various directions. The length of each microchannel was 200 μm. Microchannels were compared in six directions; (1) from left to right, (2) from right to left, (3) a horizontal reciprocating motion from left to right and then right to left, (4) from top to bottom, (5) from bottom to top, (6) a vertical reciprocating motion from top to bottom, then bottom to top. Bar, 50 μm. (**b**) Mean and standard deviation of the width distribution of the fabricated agarose channels of (1)–(6) in (**a**). The widths of these microchannels were measured at 40 different points in each line.

In the experiment, 200 μm-length microchannels were fabricated by the vertical or horizontal movements of the motorized x-y stage while applying the 90 V, 1 kHz AC voltage to the microcapillary tube. The microcapillary tube was set horizontally from left, and hence micro ionic flow was from left to right direction. Figure 2a shows the fabricated microchannels as follows; (1) from left to right (same as the micro ionic flow direction), (2) from right to left (against the micro ionic flow direction), and (3) a reciprocating motion from left to right, then from right to left, and also vertically, (4) from top to bottom (perpendicular to the micro ionic flow direction), (5) from bottom to top, and (6) a reciprocating motion from top to bottom, then from bottom to top. Figure 2b shows the summary of the fabricated widths of various directions of Figure 2a. In the graph, mean ± SD of the widths of the fabricated microchannels at 40 points in each line of (1)–(3) were 12.1 ± 0.211 μm, 11.9 ± 0.206 μm, and 12.2 ± 0.200 μm, respectively. The widths of (4)–(6) were also 13.5 ± 0.263 μm, 13.7 ± 0.242 μm, and 15.0 ± 0.613 μm, respectively. In general, the stability of fabrication width both in horizontal and vertical directions was within a few percent. Regarding the etched width at the 90 V, 1 kHz AC voltage, there is no significant difference between the one-way and a reciprocating etching in horizontal movement (parallel to the micro ionic flow direction) as shown in (1)–(3). In (4) and (5), the width of one-way movement in the vertical direction was slightly larger than the fabrication in the horizontal direction. And the width of the vertical reciprocating movement was marginally larger than the one-way movements. These increases in widths might be caused by the direction of micro ionic flow direction was perpendicular to the microchannel formation direction.

As shown in the above results, there was no significant difference in the etched widths regardless of the etching direction against the direction of micro ionic current as far as we can neglect one micrometer range differences. Hence, we have concluded the micro ionic current method does not need to consider the microcapillary setting direction for the fabrication of microstructures. When we need to fabricate more detailed designs of less than one-micrometer resolution, the difference of etching width in horizontal movement and vertical movement should be considered.

**Figure 3:**
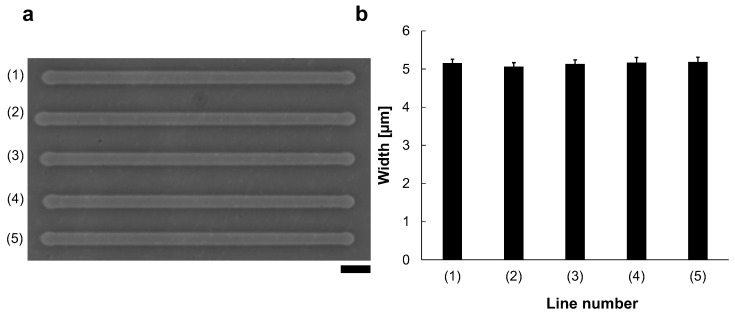
Reproducibility of the micro ionic current method for microchannel fabrication. (**a**) Micrograph of five microchannels etched to the thin agarose layer with 90 V, 1 kHz micro ionic current method. Bar, 20 μm. (**b**) Width distribution of five microchannels.

Reproducibility was also evaluated (Figure 3). We fabricated five microchannels from left to right with 90 V, 1 kHz micro ionic current, which is just the same manner as Figure 2a (1). Figure 3a shows the micrograph of the lined-up five microchannels with the same condition. The mean widths and S.D. of 40 points measurement in each line for those five lines. The results showed that (1) 5.2 ± 0.10 μm, (2) 5.1 ± 0.11 μm, (3) 5.1 ± 0.12 μm, (4) 5.2 ± 0.13 μm, and (5) 5.2 ± 0.13 μm (Figure 3b). As shown in this result, this method has high reproducibility and stability.

**Figure 4:**
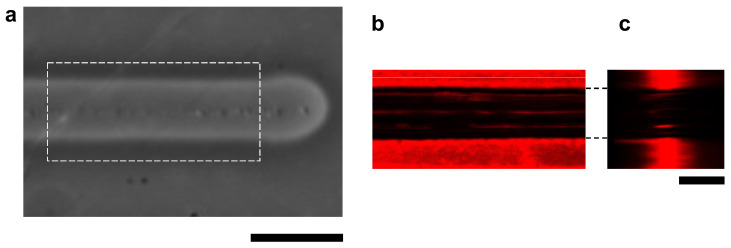
Confocal fluorescence cross-sectional image of agarose microchannel. (**a**) Bright-field microscopy image of an agarose microchamber with 100 V, 1 kHz micro ionic current and 10 μm/s stage movement. (**b**) Cross-sectional X-Y plane image of fluorescence-stained agarose layer of the dashed line box area in (**a**). (**c**) Cross-sectional X-Z plane image of fluorescence-stained agarose layer. Bars, 20 μm.

The cross-sectional structure of the microchannel was also observed using the confocal microscopy system. For the fluorescence imaging, the agarose layer was stained with 0.05 μm fluorescent microspheres. Figure 4 shows the images of a microchannel with 100 V, 1 kHz micro ionic current and 10 μm/s stage movement. As shown in Figure 4c, the sidewalls of the microchannel were perpendicular to the bottom surface of the cultivation dish.

**Figure 5:**
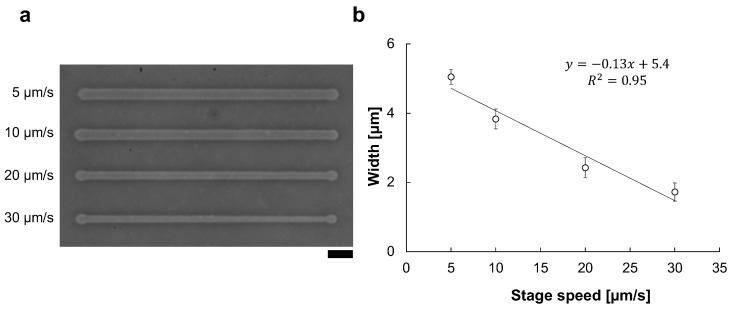
Stage velocity dependence of the micro ionic current microfabrication. (**a**) Micrograph of microchannels etched to the thin agarose layer with 90 V, 1 kHz micro ionic current method in various stage movement velocities. Bar, 20 μm. (**b**) Relationship of microchannel width and stage movement speed.

For the reproducibility of microfabrication width, precise control of stage movement speed is essential. For example, Figure 5 shows the existence of the stage velocity dependence of microchannel width change even under the same 90 V, 1 kHz condition. As shown in Figure 5b, the microchannel width and the stage speed have a directly proportional relation in the micro ionic current method. Hence, the motorized x-y stage is essential for the micro ionic current microfabrication.

### 3.3. Applied Voltage Dependence of Micro Ionic Current Microfabrication

According to Joule’s law, the quantity of heating is proportional to the voltage squared as described in Equation (1). Because the principle of agarose microfabrication is thermal melting of agarose, it is suggested that the width of the fabricated microchannel is proportional to the applied voltage squared, which makes it possible to control the processing width precisely by using voltage value. Therefore, we examined a micro ionic current method to control the widths of microchannels between the minimum and maximum voltage to fabricate the desired width.

**Figure 6:**
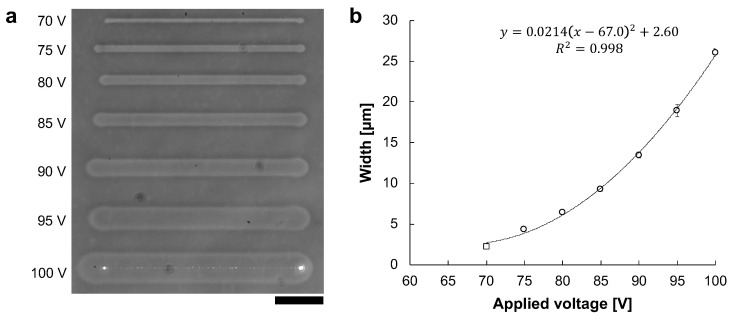
The relationship between AC voltage and microchannel width. Microchannels were fabricated with the various amplitude of 1 kHz AC voltages. The movement speed of the motorized stage was 10 μm/s. (**a**) Phase-contrast image of fabricated agarose microchannels from 70 to 100 V, respectively. Bar, 50 μm. (**b**) The relationship between applied voltage and agarose microchannel width. The widths of these microchannels were the averages of the five points in each microchannel. Open circles indicate the fabrication in which the agarose has melted sufficiently to expose the dish’s bottom surface. In contrast, an open square indicates the fabrication in which the agarose layer was melted, whereas it was not sufficient to reach the bottom surface of the cultivation dish. The width of microchannels fits well to the voltage squared, which satisfies Joule’s law.

The microchannels were fabricated with various 1 kHz AC voltages. The applied voltages were 70 V to 100 V in 5 V steps. Figure 6a shows the phase-contrast image of microchannels fabricated at voltages ranging from 70 V to 100 V. At 70 V, only the agarose surface was melted and was not reached the bottom surface of the cultivation dish. In contrast, from 75 V to 100 V, the whole agarose layer was melted to the dish surface. Figure 6b shows the relationship between applied voltage and microchannel width. The widths of microchannels fabricated at applied voltages were measured 5 points in each line, and were 2.32 ± 0.182 μm at 70 V (open square), 4.39 ± 0.240 μm at 75 V, 6.44 ± 0.213 μm at 80 V, 9.33 ± 0.276 μm at 85 V, 13.5 ± 0.357 μm at 90 V, 18.9 ± 0.734 μm at 95 V, and 26.1 ± 0.490 μm at 100 V (open circles), respectively.

The width of the fabricated microchannel was proportional to the applied voltage squared ($R^{2}=0.998$). Therefore, we can conclude that the desired width of microchannel can be fabricated by controlling the applied voltage squared effectively.

**Figure 7:**
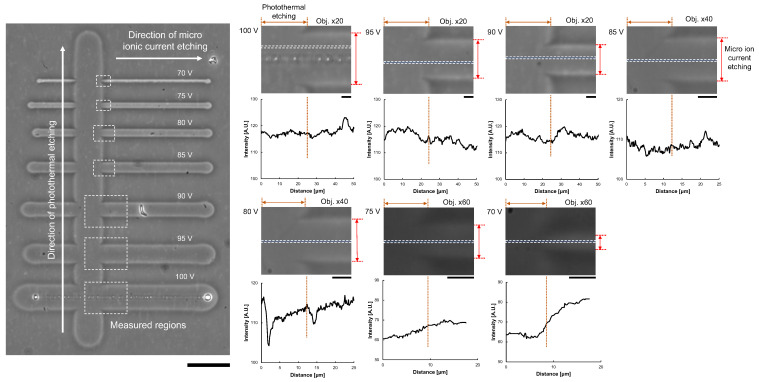
Confirmation of agarose layer removal from image analysis of agarose microchannels. Left micrograph: After the formation of agarose microchannels with micro ionic current etching as described in Figure 6a, photothermal etching was added perpendicular to the microchannels. The difference of image intensities of the micro ionic current etching region and the photothermal etching region was compared in each border of the crossing area of two etchings (dashed line box). Bar, 50 μm. Right micrographs and intensity profile graphs: Magnified micrographs and intensity profile graphs of microchannels from 70 V to 100 V micro ionic current. Intensity profiles were acquired at the dashed boxes in the magnified micrographs. The left half side of the graphs represents the intensity profile of photothermal etching, and the other right side represents micro ionic current etching. Bars, 5 μm.

To confirm the removal of the agarose layer, we compared the difference in image intensities of micro ionic current etching and photothermal etching. As photothermal etching was already used for cell cultivation, we considered the agarose layer at the photothermal etching area was removed entirely. As shown in Figure 7, after a series of microchannels was fabricated with different applied voltages of micro ionic currents, photothermal etching was added perpendicular to the microchannels. Then, the difference of intensity profiles at the border of two etchings was compared. The results showed the difference in intensities were negligible small except for 70 V micro ionic current etching.

### 3.4. AC Frequency Dependence of Micro Ionic Current Microfabrication

Just like in electric circuits, the impedance of the electrolyte solution is affected by the frequency of applied AC voltage. Therefore, we need to check the difference of ionic current against the electric current. For example, it has been reported that NaCl solution has a characteristic of decreasing impedance as the frequency increases [18]. Thus, we investigated the relationship between the width of the processed microchannel and the applied frequency under a fixed voltage.

**Figure 8:**
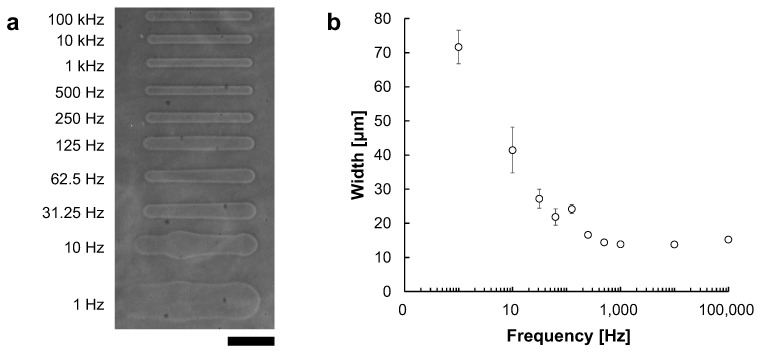
The relationship between AC frequency and microchannel width. Microchannels were fabricated with 90 V AC voltage. The movement speed of the motorized stage was 10 μm/s. (**a**) Phase-contrast image of fabricated agarose microchannels with 1–10^5^ Hz AC voltages, respectively. Bar, 100 μm. (**b**) The relationship between AC frequency and microchannels width at 90 V. The applied frequency was plotted in the logarithmic scale at X-axis. In lower frequency, the effect of peak voltage became larger, and the channel width became thicker and fluctuated. In contrast, the width became thinner in higher frequencies, and fluctuation disappeared.

Microchannels were fabricated at fixed applied voltages of 90 V with frequencies ranging from 1 Hz to 100 kHz. Figure 8a shows the phase-contrast image of microchannels fabricated at frequencies ranging from 1 Hz to 100 kHz. Figure 8b shows the relationship between frequency and microchannel width. The widths of microchannels fabricated at each frequencies were 71.6 ± 4.91 μm at 1 Hz, 41.5 ± 6.70 μm at 10 Hz, 27.2 ± 2.78 μm at 31.25 Hz, 21.9 ± 2.42 μm at 62.5 Hz, 24.2 ± 1.34 μm at 125 Hz, 16.7 ± 0.392 μm at 250 Hz, 14.4 ± 0.423 μm at 500 Hz, 13.9 ± 0.264 μm at 1 kHz, 13.8 ± 0.411 μm at 10 kHz and 15.8 ± 0.657 μm at 100 kHz. The widths were measured at 5 points in each microchannel.

As shown in the results, at lower frequencies, the channel widths became larger and fluctuated. In contrast, at higher frequencies, especially beyond 1 kHz, the widths became smaller and more stable with reaching to the asymptote line, 13 μm. These results suggest that a higher frequency is desirable for forming stable microchannels. Hence, we adopted 1 kHz AC voltage for micro ionic current microfabrication.

### 3.5. Spatial Temperature Distribution Caused by Joule Heating of Micro Ionic Current in Capillary Tube

We also have examined the spatial distribution of Joule heating in the tapered microcapillary tube using the fluorescence quenching method with rhodamine B (a temperature-dependent fluorescent dye). In principle, as fluorescence of the dye decays as the temperature rises, known as fluorescence quench [19], relative temperature distribution was estimated from the decay of fluorescence intensity with micrometer resolution [20,21].

**Figure 9:**
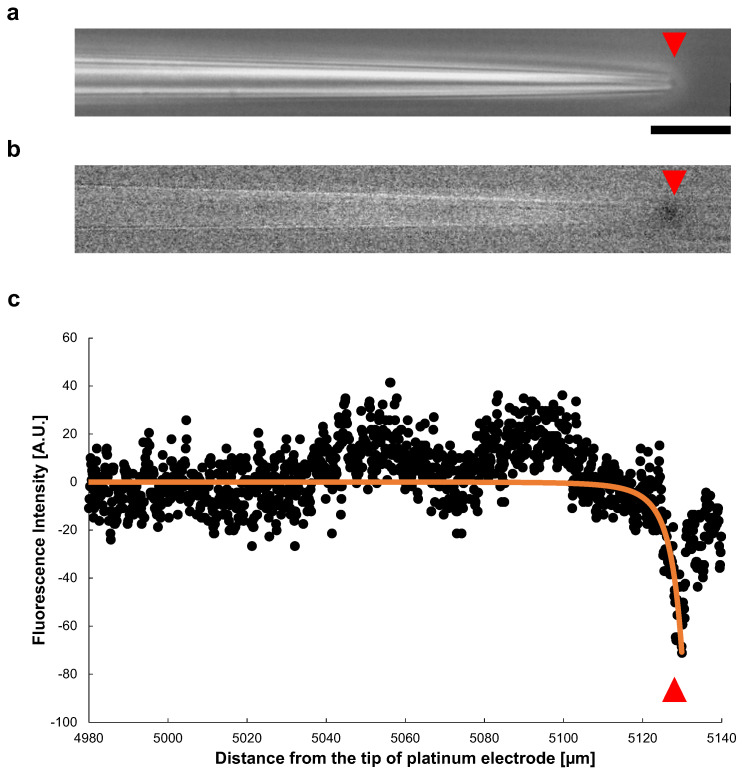
Spatial distribution of Joule heat in a tapered glass microcapillary tube. (**a**) Phase-contrast image of a microcapillary tube under AC voltage of 140 V, 1 kHz. The inner diameter of the tip was 2.7 μm. The red triangles indicate the position of the microcapillary tube tip. (**b**) Fluorescence image of (**a**). The fluorescence intensity decrease represents the temperature rise caused by the fluorescence quenching, which was observed at the tip of the microcapillary tube specifically. (**c**) Spatial distribution of fluorescence intensity in the microcapillary tube (*x*-direction). The sharp decrease in fluorescence intensity occurred at the tip of the tapered microcapillary tube. The orange line was the fitted curvature by calculating the heat value P(x) applying Equation (5), with a capillary tube inner diameter $D_{0}=1150$ μm, tip inner diameter $D_{tip}=2.7$ μm, and tapered section length, 5130 μm. Bar, 20 μm.

Figure 9a shows a phase-contrast image of a microcapillary tube during the application of a 140 V, 1 kHz AC voltage. The inner diameter of the open end was 2.7 μm. In Figure 9b, also shows the change of the fluorescence intensity after the voltage applied. A significant decay of the fluorescence intensity of Rhodamine B was observed at the tip of the tube. Figure 9c shows the spatial distribution of the fluorescence intensity change in the microcapillary tube (*x*-direction). The plots show the intensity of fluorescence at each point in the tube. The intensity decreased sharply at the tip of the tapered microcapillary tube caused by fluorescence quench, suggesting Joule heating occurred at the open end of the tapered tube.

When the inner diameter of tapered micro capillary tube at the position *x* from the platinum electrode position (x=0) is described as,

(4) $$D(x)\;=D_{0}-kx,$$

where *k* is decrement index of the tapered micro capillary tube. Hence, the spatial distribution of Joule heat becomes,

(5) $$P\left(x\right)={\left(\frac{D_{0}}{D(x)}\right)}^{4}I_{0}^{2}R={\left(\frac{1}{1-\frac{k}{D_{0}}x}\right)}^{4}I_{0}^{2}R.$$

This equation can explain the sharp and local temperature rise at the tip of the microcapillary tube even in the constant tapered microcapillary tube, which was well fit to the experimental result.

The P(x) at each point, obtained by Equation (5) was changed to a negative value to meet to fluorescence quenching results, and plotted as orange line in Figure 9c. For the fitting to Equation (5), we adopted a microcapillary tube inner diameter $D_{0}=1150$ μm, tip inner diameter $D_{tip}=2.7$ μm, and tapered section length, 5130 μm. It showed a sharp decrease at the tip of the tube, well fit to the measured value.

### 3.6. Difference between Micro Ionic Current Method and Photo-Thermal Etching Method for Agarose Microfabrication on MEA Chips

The newly developed micro ionic current method is a simple system set-up requiring only an AC function generator and an amplifier. It does not require special equipment such as laser optics in the conventional photothermal microfabrication method. Especially, as this micro ionic current method is similar to the patch-clamp set-up, this system can be added to the standard patch-clamp devices without any particular additional setting except for the high voltage AC power supply.

As this method is free from the optical index and its limitation, the advantage of this method should appear during the microfabrication of the MEA chip. When we use the conventional 1480-nm photo-thermal method, the widths of the fabricated micropatterns differed depending on the absorbance of the substrate. The absorbance of the MEA chip differed significantly according to the presence or absence of ITO wiring on the chip surface [22,23].

**Figure 10:**
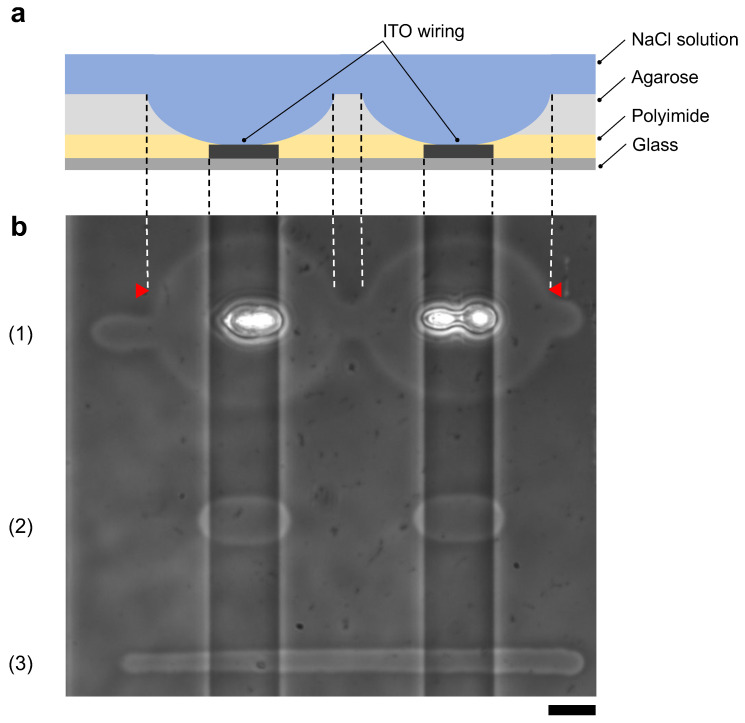
Comparison of agarose microfabrication methods on the multi-electrode array (MEA) chip. (**a**) Schematic drawing of the cross-sectional image of the MEA chip after agarose microfabrication. Indium-tin-oxide (ITO) wiring was placed on a glass substrate, and the entire surface was covered with a polyimide insulating film. The MEA chip was coated with agarose and filled with the cultivation medium. The photothermally etched region of the agarose layer corresponds to the area between two red triangles in (**b**), indicating that the polyimide insulating film was also damaged. (**b**) Phase-contrast image of agarose microchannels formed on an MEA chip with photothermal method ((1) and (2)) and micro ionic current method (3). The two vertical ITO wiring lines cross through the image. The microchannels were fabricated by the following procedures; (1) 1480-nm focused photothermal etching in high laser intensity (71 mW) to form 10 μm width microchannel on the glass substrate region, (2) 1480-nm photothermal etching in low laser intensity (30 mW) not to damage polyimide layer and (3) micro ionic current method to form a 10 μm width microchannel (130 V, 1 kHz AC voltage). In (1), the intensity of the infrared laser was set to form a 10 μm width microchannel on the glass substrate. In this intensity, a focused infrared laser formed a huge hole in the ITO region and damaged the polyimide layer by ITO absorption heating. In (2), when the applied focused beam intensity decreased not to damage polyimide, the microchannel was not fabricated on the glass substrate region because of the insufficient intensity to melt agarose. In (3), a uniform width microchannel was formed on the chip regardless of the ITO wiring. Bar, 20 μm.

Figure 10a shows schematic drawing of the cross-sectional image of the agarose coated MEA chip after photothermal etching. This MEA chip has two vertical ITO wirings on the bottom surface and polyimide was coated entire surface of the chip as an insurating film.

Figure 10b shows the results of agarose microfabrication with photothermal etching and micro ionic current microfabrication. First, using our photothermal etching system [11], we etched an agarose microchannel passing through the two ITO wire regions with photothermal microfabrication procedure. In this example, we set the intensity of 1480-nm infrared laser to 71 mW for the 10 μm width line on the glass substrate region (11.9 ± 1.22 μm). However, when the focused laser reached to the ITO region, agarose melted up to 71.8 ± 1.88 μm, which was 6.03 times wider than the glass region, and polyimide film was damaged due to ITO absorption (Figure 10(b1)). Next, we set the intensity of focused laser power to 30 mW, which dose not give any damage on polyimide film, and found that agarose was melted 18.2 ± 1.01 μm only in the ITO regions and was not melted in the glass region (Figure 10(b2)). In above experiments, the power of focused infrared laser were measured by power meter (FM 33-0506, Coherent, Inc., Santa Clara, CA, USA). The mean and SD values were acquired from five points of each width. In contrast, when we etched an agarose layer with micro ionic current method with 130 V, 1 kHz AC voltage, the width of microchannels was 8.20 ± 0.719 μm in the glass region, and 8.45 ± 0.629 μm in the ITO wiring region (Figure 10(b3)). The mean and SD values of the width was acquired from the measured 10 points.

As expected, the results showed that the micro ionic current method was able to fabricate uniform microstructures independent of absorption change of substrate such as ITO wiring.

### 3.7. Cell Cultivation in Agarose Microchambers Fabricated with Micro Ionic Current Method

Finally, we check the ability of micro ionic current agarose microfabrication for cell cultivation as the remaining issue to evaluate this method. We placed cardiomyocytes in the 20 μm microchambers fabricated with the micro ionic current method. We evaluated the influence of this method on the thin collagen layer coated just on the glass substrate and under the agarose layer for cell adhesion caused by this method.

**Figure 11:**
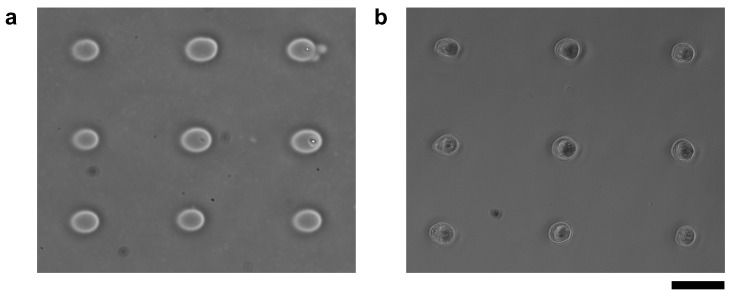
Cardiomyocytes seeded in microchambers fabricated by Joule heating of micro ionic current. (**a**) Phase-contrast image of microchambers fabricated with 90 V, 1 kHz AC voltage. The diameter of microchambers was set to 20 μm each. (**b**) Phase-contrast image of nine mouse primary cardiomyocytes after a day of cultivation started in microchambers. The cardiomyocytes adhered to the exposed bottom layer in the microchambers and started beating. Bar, 50 μm.

For this experiment, we prepared a collagen-coated cultivation dish and fabricated 20 μm microchambers by micro ionic current method with 90 V, 1 kHz AC voltage as shown in Figure 11a. Cardiomyocytes were also pre-cultured for two days, treated with trypsin-EDTA, and washed, and then nine cardiomyocytes were placed one by one in the microchambers using a manipulator. The micrograph of cardiomyocytes one day after seeding is shown in Figure 11b. The cardiomyocytes adhered to the collagen-coated bottom surface of the dish and were beaten.

**Figure 12:**
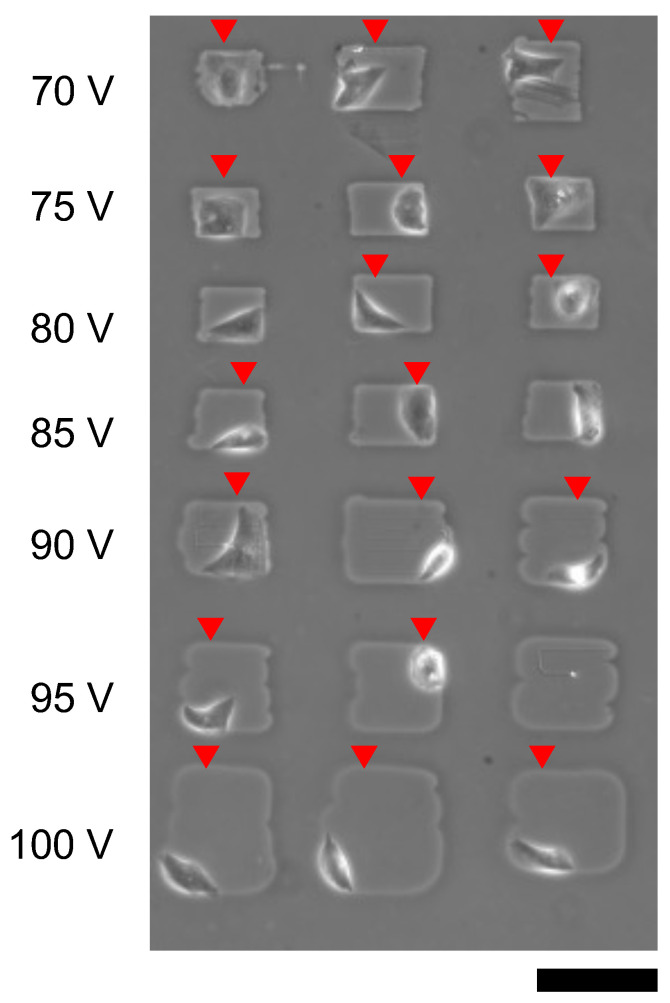
Cardiomyocyte cultivation in microchambers fabricated by different micro ionic current etching. Microchambers were formed with different voltages of micro ionic currents from 70 V to 100 V and 1 kHz, and single cardiomyocytes was placed each microchanber. The micrograph was acquired a day after cultivation started. Red arrowheads indicate spontaneusly beating cardiomyocytes. Bar, 100 μm.

Figure 12 also shows the single cardiomyocyte cultivation of microchambers formed from 70 V to 100 V, 1 kHz micro ionic currents. The results showed cardiomyocytes were attached on the bottom surface of the collagen-coated layer even 70 V microfabrication, which was expected to be insufficient to remove the agarose layer as described above. And the spontaneous beating of cardiomyocytes was observed in 18 microchambers.

These results indicate that the local heating by micro ionic current did not affect collagen, which was an adhesion factor, and cardiomyocytes were cultivated in microchambers effectively. Then, the remaining issue is the effect of additional stepwise microfabrication of micro ionic current method to the cultivated cells, especially neurons and neurites.

**Figure 13:**
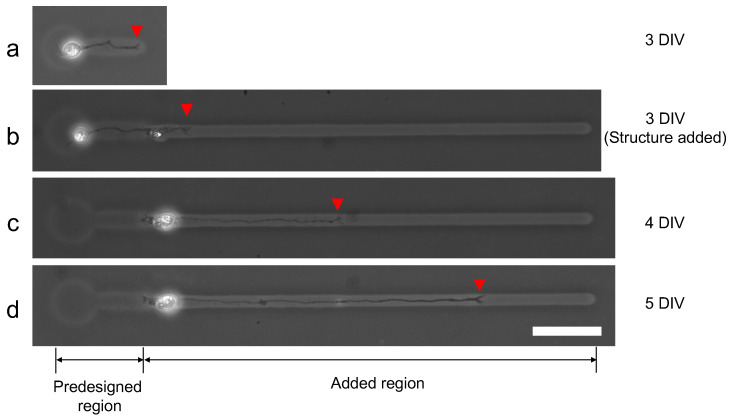
Stepwise microfabrication to guide elongated neurite. (**a**) Single neurite elongated in the prefabricated microchannel (three days after cultivation started (3DIV)). (**b**) After the observation of (**a**), an additional microchannel was fabricated from the end of the existing microchannel with 100 V, 1 kHz micro ionic current etching. The micrograph was captured two hours after the additional microfabrication. (**c**) A day after an additional microchannel was fabricated (4DIV). The neurite elongated into the added region of the microchannel. (**d**) Two days after additional fabrication (5DIV). The neurite continued to elongate in the added region of the microchannel. Red arrowheads represent the leading edge of the elongated neurite. Bar, 50 μm.

We evaluated the ability of additional microfabrication during single neuron cultivation. Figure 13 shows the result of single neurite elongation after additional microchannel fabrication with 100 V, 1 kHz micro ionic current method. Three days after cultivation started (3DIV), a single neurite elongated into the predesigned microchannel and reached the end of the channel (Figure 13a). Then the additional microchannel was added to the end of the existing microchannel, and the elongation was observed two hours after re-cultivation started (Figure 13b). A day after re-cultivation started, the neurite elongated to the added region of the microchannel (Figure 13c) and continued even in the next day (Figure 13d). The results also support the evidence that this micro ionic current method can be used for additional microfabrication, even for stepwise single neurite elongation guiding.

### 3.8. Advantages and Potential Improvements of Micro Ionic Current Method

As described above, there are three advantages identified for this agarose microfabrication method utilizing Joule heat of micro ionic current. The first is the simple system set-up. Unlike the conventional photothermal agarose microfabrication method, this system does not require the laser sauce and the laser optics, which require expertise, especially for invisible infrared laser focusing. Secondly, the system can fabricate thin and uniform 3 to 20 μm microchannels independent of the diffraction index, which limits the minimum focusing area up to the range of wavelength of infrared light. The width of the linear pattern can be fine-tuned depending only on the applied voltage and is stable as far as the voltage is kept constant. Third and finally, the substrate material does not affect the fabrication of agarose. As the conventional photothermal agarose microfabrication technology applied absorbance of a focused infrared laser to convert to heat for etching agarose, it was impossible to create a uniform line pattern on the patterned substrate such as the MEA chip. Even though the permeable ITO wiring was used for the MEA chip, its absorbance to infrared laser formed unexpected and undesired intense heat when the focused beam was absorbed in the ITO region. However, with this new technology, Joule heat of micro ionic current does not influence by the local difference of absorbance of materials on the substrate, and hence, making it possible to create uniform width line patterns even on the ITO wiring chip surface.

While this new technology has many advantages against the photothermal method, it still has room to improve its potential abilities for the next step. In this paper, we used 2 μm-open-end microcapillary tube for etching agarose layer to form the micrometer-sized structures. However, in principle, as this method is independent of the optical or physical size limitation, it must have the ability to create smaller micro ionic currents to form structures much smaller than a micrometer range. The applied voltage, a sauce of ionic current at the tip of the microcapillary tube, is also a candidate for improvement. In this experiment, we set a pair of electrodes in a microcapillary tube, and the buffer of the cultivation dish as the ground line, and long-distance of high impedance material such like cultivation buffer requires a higher voltage to acquire a sufficient amount of ionic current for Joule heating. So if the distance of these two electrodes can be minimized, the required amplitude of voltage should become a magnitude smaller. We also should consider the mobility of carrier ions to generate higher Joule heating effectively. In contrast to the current in the electric circuits, the divalent ions and trivalent ions can form the two-times or three-times larger currents only by replacing the current carrier ions from sodium ions as far as they do not influence the cultivating condition of cells, and hence it must contribute to the reduction in the required voltage of power supply. These potentials will be examined and reported in the subsequent papers.

## 4. Conclusions

We have developed a new agarose microfabrication technology exploiting Joule heating of micro ionic current at the tip of the microcapillary tube. The results showed that the ability of this micro ionic current method is similar or even better to the conventional photothermal etching technology. Its several advantages overcome the remaining potential limitation of the photothermal etching approach. Since we have developed and improved agarose microfabrication technology since 2002, we applied it for constructive cell network studies in cardiomyocytes, neurons, and epithelial cells. Applying this new approach, as we introduced in this paper, agarose microfabrication technology can be used for more potential cell-network studies without preparing an expensive photothermal infrared laser set-up. As the next step, the remaining potential limitations of this method will be examined and be solved with our next stage of improvements.

## Data Availability

Not applicable.
